# LINC00514 promotes gastric cancer cell growth and EMT progression via miR-204-3p/KRAS

**DOI:** 10.18632/aging.202905

**Published:** 2021-04-22

**Authors:** Ling Yuan, Jiaxin Li, Yi Yang, Yan Chen, Yang Bu, Mengyi Ye, Xiongjie Mao, Tingting Ma, Lei Yu, Yi Nan

**Affiliations:** 1 Pharmacy College of Ningxia Medical University, Yinchuan 750004, China; 2 Ningxia Medical University Key Laboratory of Hui Ethnic Medicine Modernization Ministry of Education, Yinchuan 750004, China; 3 Traditional Chinese Medicine College of Ningxia Medical University, Yinchuan 750004, China; 4 Hepatobiliary Surgery, General Hospital of Ningxia Medical University, Yinchuan 750004, China; 5 Department of Infectious Disease, The Fourth Hospital of Harbin Medical University, Harbin 150001, Heilongjiang, China

**Keywords:** LINC00514, KRAS, miR-204-3p, gastric cancer

## Abstract

Long noncoding RNAs (LncRNAs) participate in tumor development and tumorigenesis. However, the mechanism, function and expression of LINC00514 in GC remain unknown. We showed that LINC00514 was upregulated in GC specimens compared with nontumor specimens. Overexpression of LINC00514 induced cell growth and EMT progression in GC cells. By using bioinformatics prediction, we found that miR-204-3p contained binding sequences for LINC00514. Luciferase reporter analysis noted that miR-204-3p overexpression decreased the luciferase expression under LINC00514-wild-type and KRAS-wild-type reporters but not that under mutant reporter. Ectopic LINC00514 expression decreased miR-204-3p expression. miR-204-3p expression was decreased in GC specimens compared with nontumor specimens and that LINC00514 was negatively correlated with miR-204-3p in GC specimens. Furthermore, KRAS was identified as a target gene for miR-204-3p according to TargetScan. Elevated miR-204-3p expression inhibited KRAS expression in HGC-27 cells, and ectopic expression of LINC00514 enhanced KRAS expression. Elevated LINC00514 expression enhanced cell growth and EMT progression by sponging KRAS. Our data indicated that LINC00514 may act as an oncogene and therapeutic target for GC.

## INTRODUCTION

Gastric cancer is the 2^nd^ cause of tumor-relevance death in the world [1–3]. There are about 900 thousand novel gastric cancer cases and 700 thousand deaths worldwide annually [4, 5]. Although chemotherapy and surgery reduced development of gastric tumor and inhibited tumor metastasis, the prognosis of this disease remains dissatisfied [6–9]. Gastric tumor is one heterogeneous disease which evolves in many epigenetic and genetic alterations [10–12]. Thus, it is critical to study molecular changes that influenced gastric cancer development pathways and then find new approaches for this decrease treatment and diagnosis.

LncRNAs are small transcripts which cannot encode proteins, up to about hundred kbs [13–16]. LncRNAs are deregulated in several cancers including glioma, papillary thyroid tumor, ovarian carcinoma, osteosarcoma and gastric tumor [13, 17–24]. More evidences have indicated that lncRNAs play roles in many biological procedures, such as differentiation, apoptosis, EMT, proliferation and metabolism [24–27]. Moreover, Li et al. [28] showed that downregulation of LINC00514 suppressed papillary thyroid tumor cell migration, growth and invasion. Yu et al. [29] illustrated that LINC00514 enhanced osteosarcoma development by sponging miR-708/URGCP. Mi et al. [30] also noted that SP1-influenced LINC00514 overexpression induced metastasis and growth by modulating miR-708 in osteosarcoma. Until now, the roles of LINC00514 in gastric tumor are undefined and need to be studied.

We found that LINC00514 expression was upregulated in GC specimens compared with nontumor specimens. Overexpression of LINC00514 induced cell growth and EMT progression in GC cells.

## MATERIALS AND METHODS

GC specimens and paired control nontumor samples were collected from 40 cases of GC at our department. Samples were immediately stored and snap-frozen in liquid nitrogen. Our protocol was approved by the Clinical Ethics Committee of Ningxia Medical University. HGC-27 and SGC-7901 cells were obtained from ATCC (Invitrogen, USA) and plated in DMEM supplemented with streptomycin, FBS and penicillin. PcNDA-LINC00514 and miR-204-3p mimic and control vectors were obtained from GenePharma (Shanghai, China). Cell transfection was performed using Lipofectamine 2000 (Invitrogen, USA).

### CCK-8 analysis

Cells were plated in a 96-well microplate, and cell proliferation was assessed by CCK-8 kit (Dojindo). The growth rate was detected at 0, 1, 2 and 3 days post-transfection, and the absorbance at 450 nM was detected with a microtiter reader.

### qRT-PCR assay

Total RNA was isolated utilizing TRIzol (Invitrogen, USA) from cells and specimens according to these instructions. qRT-PCR analysis was conducted to detect mRNA, miRNA and lncRNA levels on the CFX96 Bio-Rad system utilizing SYBR Mix following the manufacturer instructions. The relative expression was calculated by the 2^-ΔΔCt^ method. The primers used were: miR-204-3p, Forward, 5’-AGCTGTACAAGTAAGCCTGATCATGTACCCATAGG-3’ and Reverse, 5’-GGGAGAGGGGCTTAGCTTATGGGACAGTTATGGGC-3’. LINC00514, Forward, 5’-GCTCAACATCTCACTTCTCCCAC-3’ and Reverse, 5’-CCTTCAGTGTCTGGGAAAGAGAG-3’. GAPDH, Forward, 5’- CGGAGTCAACGGATTTGGTCGTAT-3’ and Reverse, 5’- AGCCTTCTCCATGGTGGTGAAGAC-3’. U6, Forward, 5’- GCTTCGGCAGCACATATACTAAAAT-3’ and Reverse, 5’- CGCTTCACGAATTTGCGT GTCAT-3’.

### Dual Luciferase Reporter

The mutated and wild-type putative miR-204-3p targets on LINC00514 and the KRAS 3’UTR were then cloned into the pGL3 expression vector (Invitrogen). Cells were cultured in 24-well dishes and transfected with scramble or miR-204-3p mimic and wt LINC00514 and KRAS 3’UTR or mutated LINC00514 and KRAS 3’UTR vectors. After 48 hours, luciferase values were measured with Dual-Luciferase System (Promega, USA).

### Statistical analysis

Data are indicated as the means ± SD, and statistical assays were utilized by SPSS. Statistical significance was detected using Student’s t-test. Statistical significance was measured at P<0.05.

## RESULTS

### LINC00514 was overexpressed in GC specimens

**Figure 1:**
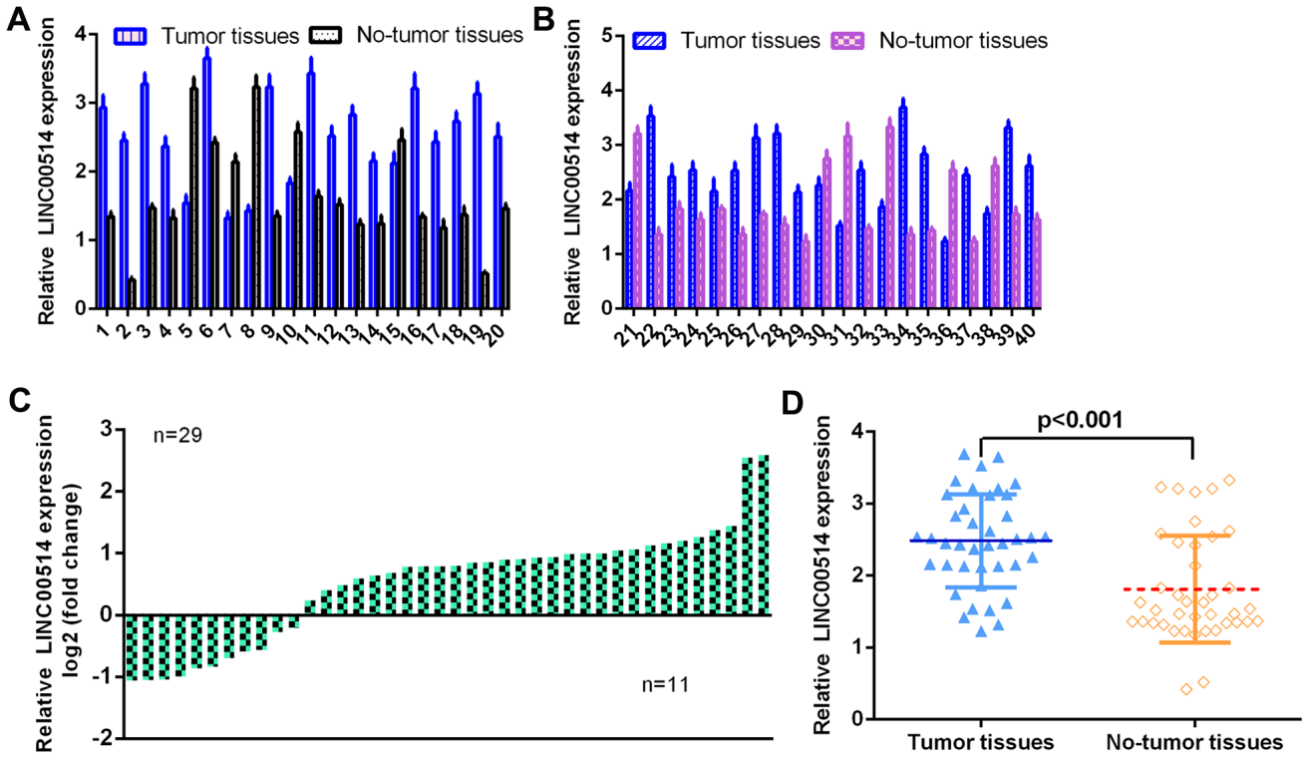
**LINC00514 was overexpressed in GC specimens.** (**A**, **B**) The expression of LINC00514 was detected in 40 pairs of GC specimens and paired control nontumor specimens using qRT-PCR. (**C**) The expression of LINC00514 was overexpressed compared to paired control nontumor specimens in 29 cases (29/40, 72.5%). (**D**) LINC00514 expression was upregulated in GC specimens compared with nontumor specimens.

First, the level of LINC00514 was detected using qRT-PCR in 40 pairs of GC specimens and paired control nontumor specimens. As illustrated in Figure 1A, 1B, the expression of LINC00514 in 40 pairs of GC specimens and paired control nontumor specimens is indicated. LINC00514 was overexpressed in 29 cases (29/40, 72.5%) compared to paired control nontumor specimens (Figure 1C). LINC00514 expression was upregulated in GC specimens compared with nontumor specimens (Figure 1D).

### miR-204-3p was decreased in GC specimens

**Figure 2:**
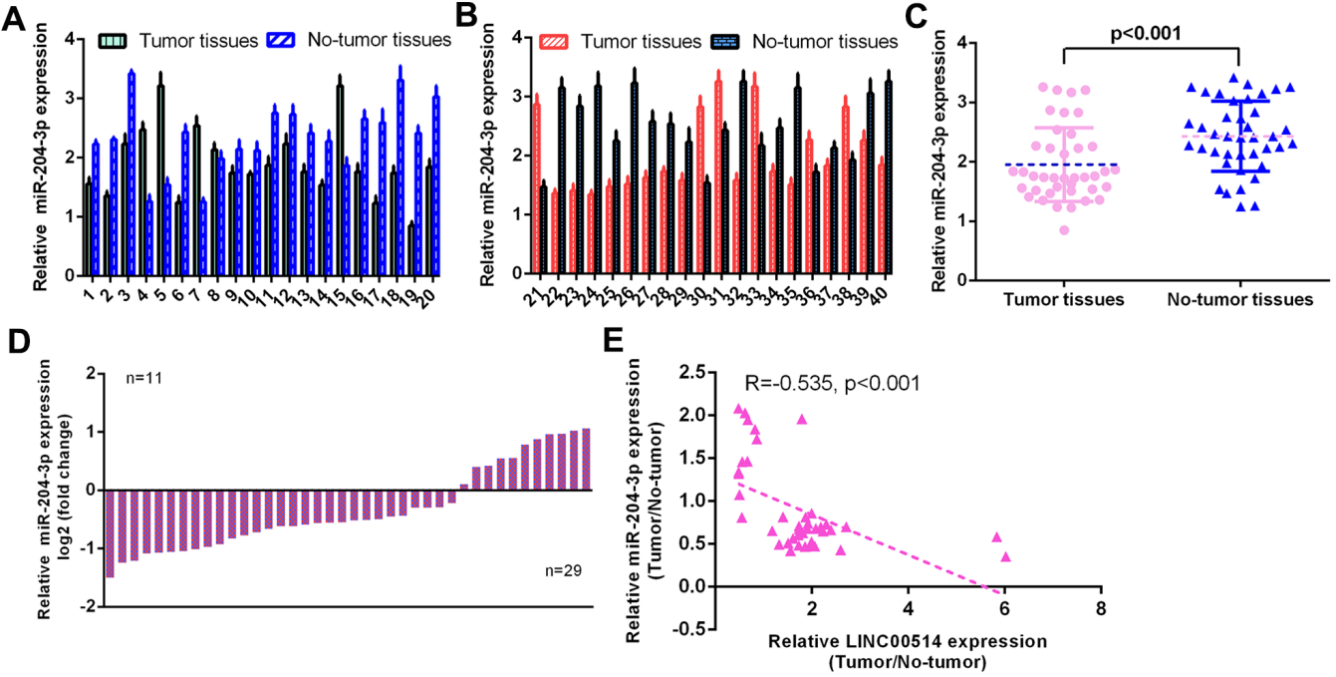
**miR-204-3p was decreased in GC specimens.** (**A**, **B**) The expression of miR-204-3p was detected in 40 pairs of GC specimens and paired control no-tumor specimens using qRT-PCR. (**C**) The expression of miR-204-3p was decreased in 29 cases (29/40, 72.5%) compared to paired control no-tumor specimens. (**D**) The miR-204-3p expression was downregulated in GC specimens compared with no-tumor specimens. (**E**) LINC00514 expression was negatively correlated with miR-204-3p expression in GC specimens.

Then, the level of miR-204-3p was determined using qRT-PCR in 40 pairs of GC specimens and paired control nontumor specimens. The expression of miR-204-3p in 40 pairs of GC specimens and paired control nontumor specimens is illustrated in Figure 2A, 2B. The expression of miR-204-3p was decreased compared to paired control nontumor specimens in 29 cases (29/40, 72.5%) (Figure 2C). miR-204-3p expression was downregulated in GC specimens compared with nontumor specimens (Figure 2D). LINC00514 expression was negatively correlated with miR-204-3p expression in GC specimens (Figure 2E).

### Overexpression of LINC00514 induced cell growth and EMT progression in GC cells

**Figure 3:**
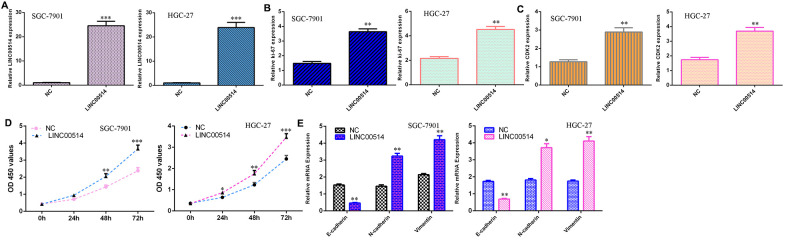
**Overexpression of LINC00514 induced cell growth and EMT progression in GC cells.** (**A**) The level of LINC00514 was overexpressed in SGC-7901 and HGC-27 cells after transfection with pcDNA-LINC00514. (**B**) Elevated expression of LINC00514 increased Ki-67 expression in both SGC-7901 and HGC-27 cells. (**C**) The expression of CDK2 in SGC-7901 and HGC-27 cells was measured by qRT-PCR. (**D**) Overexpression of LINC00514 induced cell growth in both SGC-7901 and HGC-27 cells. (**E**) Ectopic expression of LINC00514 inhibited E-cadherin expression and increased vimentin and N-cadherin expression. *p<0.05, **p<0.01 and ***p<0.001.

The level of LINC00514 was overexpressed in both SGC-7901 and HGC-27 cells after transfection with pcDNA-LINC00514 (Figure 3A). Elevated LINC00514 expression increased Ki-67 expression in both SGC-7901 and HGC-27 cells (Figure 3B). Overexpression of LINC00514 enhanced CDK2 expression in both SGC-7901 and HGC-27 cells (Figure 3C). Overexpression of LINC00514 induced cell growth in both SGC-7901 and HGC-27 cells (Figure 3D). Ectopic expression of LINC00514 inhibited expression of E-cadherin and increased vimentin and N-cadherin expression (Figure 3E).

### LINC00514 sponged miR-204-3p/KRAS expression in GC cells

**Figure 4:**
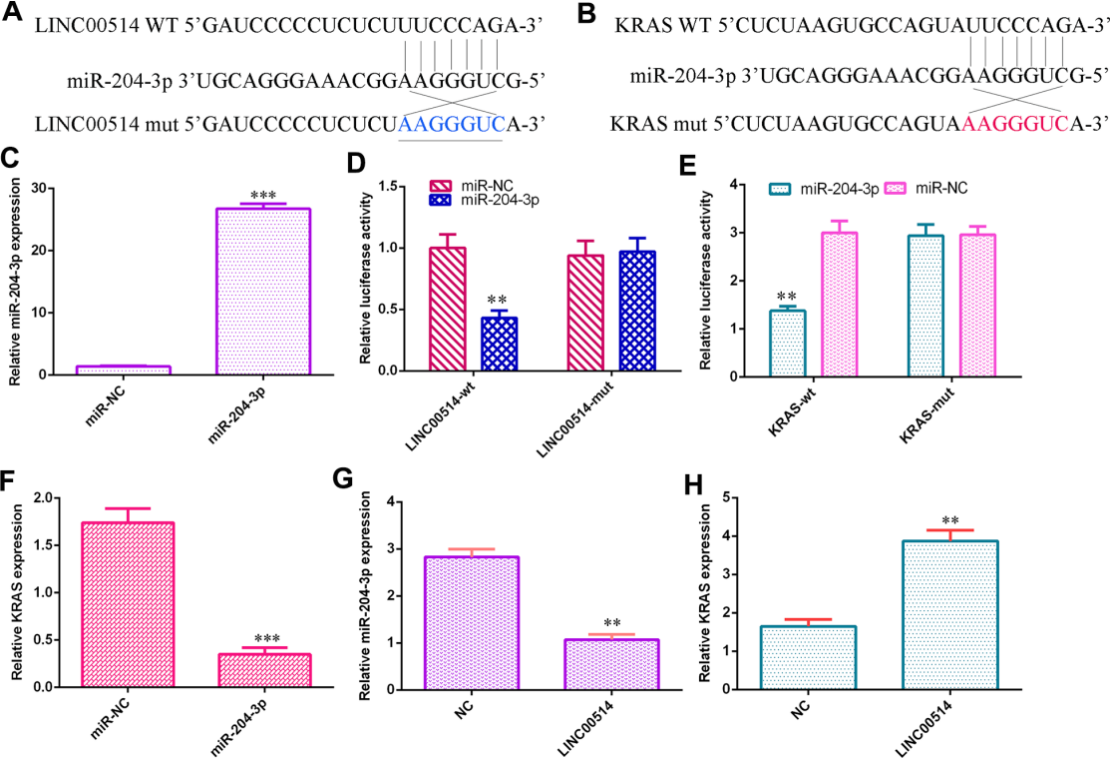
**LINC00514 sponged miR-204-3p/KRAS expression in GC cells.** (**A**) LINC00514 may be a sponge gene for miR-204-3p by utilizing the bioinformatics tools in starBase. (**B**) It was noted that KRAS was a potential target gene for miR-204-3p according to TargetScan bioinformatic analysis. (**C**) The level of miR-204-3p was upregulated in HGC-27 cells after transfection with the miR-204-3p mimic. (**D**) Overexpression of miR-204-3p decreased the luciferase value of the LINC00514-wild-type reporter but not that under the mutant reporter. (**E**) Elevated expression of miR-204-3p suppressed the luciferase value of the KRAS-wild-type reporter but not that under the mutant reporter. (**F**) Elevated expression of miR-204-3p suppressed KRAS expression in HGC-27 cells. (**G**) Ectopic expression of LINC00514 inhibited miR-204-3p expression in HGC-27 cells. (**H**) Overexpression of LINC00514 promoted KRAS expression in HGC-27 cells. **p<0.01 and ***p<0.001.

Utilizing the starBase database, we found that LINC00514 may be a sponge gene for miR-204-3p (Figure 4A). Then, using TargetScan bioinformatics analysis, it was indicated that KRAS was a potential target gene for miR-204-3p (Figure 4B). The level of miR-204-3p was upregulated in HGC-27 cells after transfection with the miR-204-3p mimic (Figure 4C). Luciferase reporter analysis illustrated that overexpression of miR-204-3p decreased the luciferase expression under LINC00514-wild-type and KRAS-wild-type reporters but not that under the mutant reporter (Figure 4D, 4E). Elevated miR-204-3p expression inhibited KRAS expression in HGC-27 cells (Figure 4F). Ectopic expression of LINC00514 decreased miR-204-3p expression (Figure 4G) and enhanced KRAS expression (Figure 4H).

### Elevated expression of LINC00514 enhanced cell growth and EMT progression by sponging KRAS

**Figure 5:**
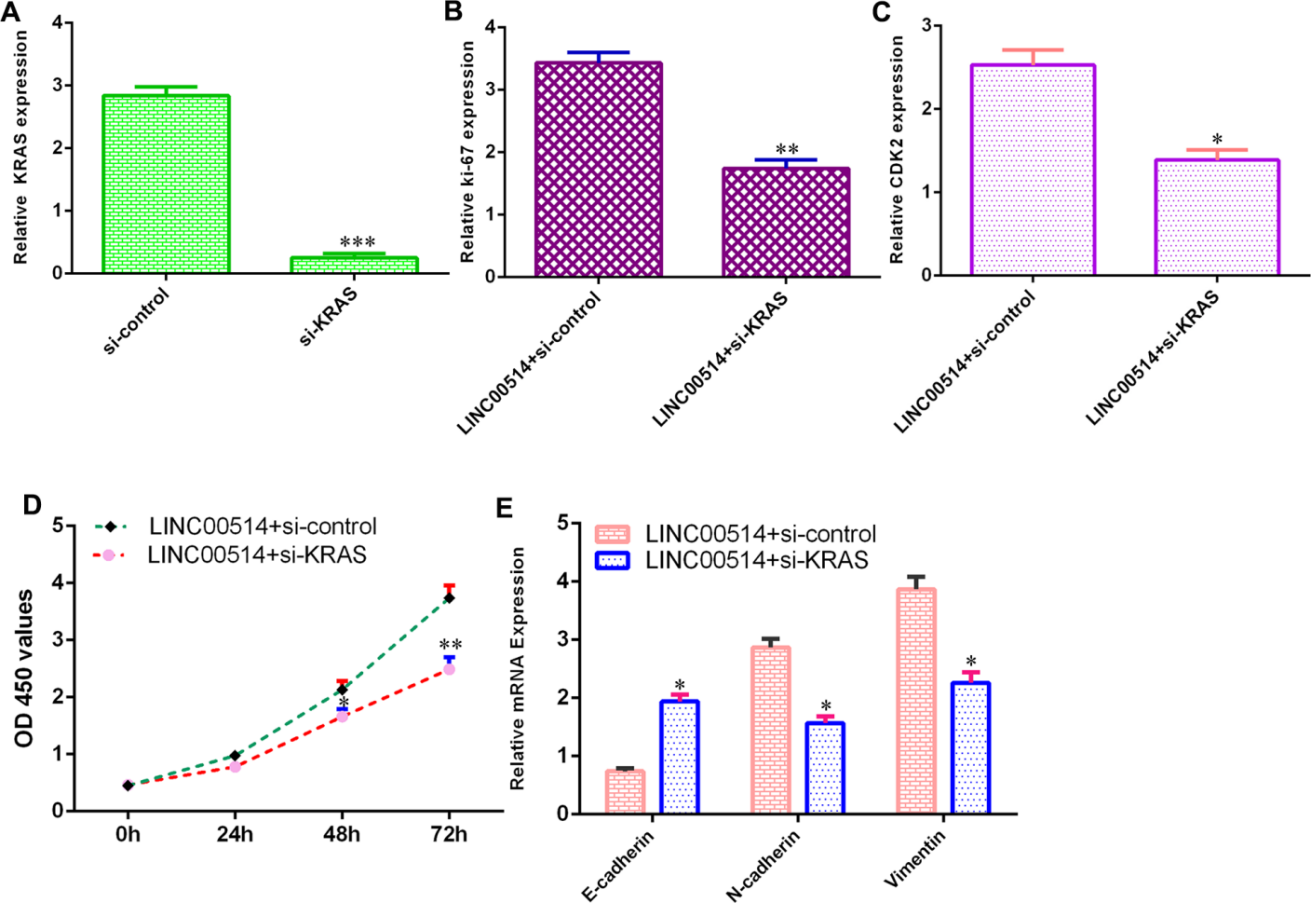
**Elevated expression of LINC00514 enhanced cell growth and EMT progression by sponging KRAS.** (**A**) The level of KRAS was downregulated in HGC-27 cells after transfection with si-KRAS vectors. (**B**) The expression of KRAS was measured by qRT-PCR. (**C**) Downregulation of KRAS suppressed CDK2 expression in LINC00514-overexpressing HGC-27 cells. (**D**) Knockdown of KRAS inhibited cell growth in LINC00514-overexpressing HGC-27 cell. (**E**) The expression levels of E-cadherin, N-cadherin and vimentin were detected by qRT-PCR. *p<0.05, **p<0.01 and ***p<0.001.

To further understand the functional roles of the LINC00514/KRAS axis in GC development, we used KRAS siRNA vectors for experiments. The level of KRAS was downregulated in HGC-27 cells after transfection with si-KRAS vectors (Figure 5A).

Downregulation of KRAS inhibited Ki-67 expression (Figure 5B) and CDK2 expression (Figure 5C) in LINC00514-overexpressing HGC-27 cells. Knockdown of KRAS inhibited cell growth in LINC00514-overexpressing HGC-27 cells (Figure 5D). Downregulation of KRAS promoted E-cadherin expression and decreased N-cadherin and vimentin expression (Figure 5E).

## DISCUSSION

LncRNAs have drawn increasing attention due to their roles in the development and progression of tumors. Many references have noted that lncRNAs play critical roles in GC development, indicating new insight into GC pathogenesis. For instance, HNF1A-AS1 induced cell angiogenesis, metastasis, invasion and lymphangiogenesis by sponging the miR-30b-3p/PI3K/AKT axis in GC [31]. Liang et al. [32] noted that LINC00691 overexpression increased the invasion and growth of GC cells through JAK/STAT signaling. Zhou et al. [33] proved that BCAR4 increased cell growth and inhibited cell apoptosis by modulating MAPK/ERK in GC. Dai et al. [34] indicated that UCA1 enhanced cisplatin resistance by inducing the PI3K/AKT signaling pathway and recruiting EZH2 in GC. Recently, Li et al. [28] noted that knockdown of LINC00514 suppressed papillary thyroid tumor cell migration, growth and invasion. Yu et al. [29] illustrated that LINC00514 enhanced osteosarcoma development by sponging miR-708/URGCP. Mi et al. [30] also noted that SP1-influenced LINC00514 overexpression induced metastasis and growth by modulating miR-708 in osteosarcoma. However, the role of LINC00514 in GC is still unknown and needs to be studied. We illustrated that LINC00514 expression was upregulated in GC specimens compared with nontumor specimens. Overexpression of LINC00514 induced cell growth and EMT progression in GC cells.

LncRNAs play a ceRNA role in sponging miRNAs and their target genes to regulate cell functions [35, 36]. For example, Wang et al. [37] illustrated that PVT1 induced GC cell migration by sponging miR-30a/Snail. Li et al. [38] noted that IGF2-AS enhanced GC cell invasion, growth and migration by regulating miR-937/EZH2. Deng et al. [39] showed that DLGAP1-AS1 induced GC progression by sponging miR-628-5p/AEG-1. Liu et al. [40] noted that SNHG1 induced GC cell EMT progression via modulation of the DCLK1/miR-15b/Notch1 axis. Recently, Li et al. [28] found that LINC00514 suppressed thyroid tumors by sponging the CDC23/miR-204-3p axis. By using bioinformatics prediction, miR-204-3p contained binding sequences for LINC00514. Luciferase reporter analysis illustrated that overexpression of miR-204-3p decreased the luciferase expression under LINC00514-wild-type and KRAS-wild-type reporters but not that under the mutant reporter. Ectopic expression of LINC00514 decreased miR-204-3p expression. We illustrated that miR-204-3p expression was downregulated in GC specimens compared with nontumor specimens and that LINC00514 expression was negatively correlated with miR-204-3p expression in GC specimens. Previous studies have shown that miR-204 plays important roles in gastric cancer development. For example, Zhang et al. showed that miR-204-5p inhibited tumor metastasis by modulating CXCR4 and CXCL12 in gastric cancer [41]. Furthermore, another study indicated that miR-204-5p suppressed gastric cancer cell growth by inhibiting RAB22A and USP47 [42]. Furthermore, KRAS was identified as a potential target gene for miR-204-3p according to TargetScan bioinformatics prediction. Elevated expression of miR-204-3p inhibited KRAS expression in HGC-27 cells, and ectopic expression of LINC00514 enhanced KRAS expression. Elevated expression of LINC00514 enhanced cell growth and EMT progression by sponging KRAS. Previous studies have shown that KRAS plays critical roles in gastric tumor development. However, the underlying mechanisms are still unclear. Our results suggested that the ability of LINC00514 to modulate KRAS may provide the mechanism of posttranscriptional regulation of KRAS.

In summary, LINC00514 was overexpressed in GC specimens, and elevated expression of LINC00514 enhanced cell growth and EMT progression by sponging the miR-204-3p/KRAS axis. Our data indicated that LINC00514 may act as an oncogene and therapeutic target for GC.
